# Huangqin-tang ameliorates dextran sodium sulphate-induced colitis by regulating intestinal epithelial cell homeostasis, inflammation and immune response

**DOI:** 10.1038/srep39299

**Published:** 2016-12-16

**Authors:** Ying Zou, Jiantao Lin, Wenyang Li, Zhuguo Wu, Zhiwei He, Guoliang Huang, Jian Wang, Caiguo Ye, Xiaoyan Cheng, Congcong Ding, Xuebao Zheng, Honggang Chi

**Affiliations:** 1 Department of Traditional Chinese Medicine, Scientific Research Platform, The Second Clinical Medical College, Guangdong Medical University, Dongguan 523808, China; 2 Sino-American Cancer Research Institute, Key Laboratory for Medical Molecular Diagnostics of Guangdong Province, Guangdong Medical University, Dongguan 523808, China; 3 Traditional Chinese Medicine and New Drug Research Institute, Guangdong Medical University, Dongguan 523808, China; 4 The Second Clinical Medical College, Guangdong Medical University, Dongguan 523808, China; 5 Mathematical Engineering Academy of Chinese Medicine, Guangzhou University of Chinese Medicine, Guangzhou, 510006, China

## Abstract

Huangqin-tang (HQT) is a traditional Chinese medicine (TCM) formula widely used for the treatment of inflammatory bowel disease in China. However, the molecular mechanisms by which HQT protects the colon are unclear. We studied the protective effects of HQT and the underlying mechanisms in an experimental mouse model and *in vitro. In vivo*, dextran sodium sulphate (DSS)-induced acute and chronic colitis were significantly ameliorated by HQT as gauged by phenotypic, histopathologic and inflammatory manifestations of the disease. Mechanistically, DSS-induced nuclear factor-κB (NF-κB) signalling was inhibited by HQT. Moreover, HQT-treated mice demonstrated significant changes in cell apoptosis, expression of apoptosis-associated genes such as caspase-3, bax, bcl-2, and intestinal permeability. HQT also increased occluding and zonula occludens-1 (ZO-1), inhibited cell proliferation (Ki67), and increased regulatory T cells numbers, protein expression of Foxp3 and IL-10 in the colonic tissue. *In vitro*, HQT down-regulated production of pro-inflammatory cytokines and supressed the NF-κB signalling pathway in lipopolysaccharides-induced RAW 264.7 macrophages. Our study suggests that HQT plays a critical role in regulating intestinal epithelial cell homeostasis, inflammation and immune response in colitis and offers novel therapeutic options in the management of inflammatory bowel disease.

Inflammatory bowel diseases (IBD), including ulcerative colitis (UC) and Crohn’s disease (CD), are a group of chronically recurring inflammatory disorders characterised by the presence of intestinal inflammation and epithelial injury and associated with an increased risk of colorectal cancer^1^,^2^. Although the precise aetiology of IBD remains largely elusive, accumulating evidence suggests that abnormal immune responses, complex gene-environment interactions and genetic factors converge to provoke disease initiation and progression^3^. It is well known that maintenance of the homeostasis of intestinal epithelial cells (IECs), which is dependent on tight regulation of proliferation and apoptosis, is a key factor in maintaining optimal gastrointestinal health^4^. A dysregulated mucosal epithelial barrier facilitates access of the luminal antigens across the epithelium, resulting in chronic intestinal inflammation and uncontrolled immune response^5^. Therefore, the restoration and maintenance of the intestinal epithelial homeostasis is critical for limiting mucosal inflammation and promoting mucosal healing, and may represent an attractive option in the management of IBD. Current therapeutic options for IBD include administration of mesalazine, immunosuppressive drugs, corticosteroids and monoclonal antibodies to tumour necrosis factor-α (TNF-α)^6^. Although these agents are effective to various extents, they are associated with serious side effects, such as increased risk of infection, malignancy, and autoimmunity^7^. Therefore, there is an urgent need for novel efficient and safe strategies for IBD management.

Traditional Chinese medicine (TCM) is based on extensive knowledge and experience gained from its widespread use over thousands of years. Thousands of prescriptions for the treatment of complex chronic diseases have been verified for efficacy and safety^8^,^9^. Huangqin-Tang (HQT) decoction is a traditional herbal formula described in the Chinese medicine book “Discussion of Cold Damage” (“Shang-Han-Lun” in Chinese) that has been used for nearly 1,800 years. It consists of 4 components: the roots of *Scutellaria baicalensis* Georgi. (skullcap, 9 g), *Glycyrrhiza uralensis* Fisch. (liquorice, 6 g), *Paeonia lactiflora* Pall. (peony, 6 g), and the fruit of *Ziziphus jujuba* Mill. (Chinese date, 6 g). In TCM, HQT is prescribed for common gastrointestinal complaints, such as diarrhoea, abdominal spasms, fever, headache, vomiting, nausea, extreme thirst and sub-cardiac distention^10^. HQT has been demonstrated to be clinically effective in the treatment of IBD. Our group and other researchers have demonstrated that HQT plays an important role in inhibiting intestinal inflammation and restoring the balance of CD4+T cells in experimental animal models with 2, 4, 6-trinitrobenzenesulfonic acid (TNBS)-induced colitis^11^,^12^. However, it is unclear whether HQT contributes to epithelial cell homeostasis and suppression of inflammation and immune response in other colitis models.

Therefore, we studied the molecular mechanism of HQT action in murine experimental models of colitis *in vivo* and assessed its anti-inflammatory effect in RAW264.7 macrophages exposed to inflammatory insults by using lipopolysaccharides (LPS) *in vitro*. Our results strongly suggest the importance of HQT in the maintenance of intestinal epithelial homeostasis and in the modulation of the inflammatory and immune response in dextran sodium sulphate (DSS)-induced colitis in mice.

## Results

### HQT attenuates colitis in a DSS-induced acute colitis model

**Figure 1:**
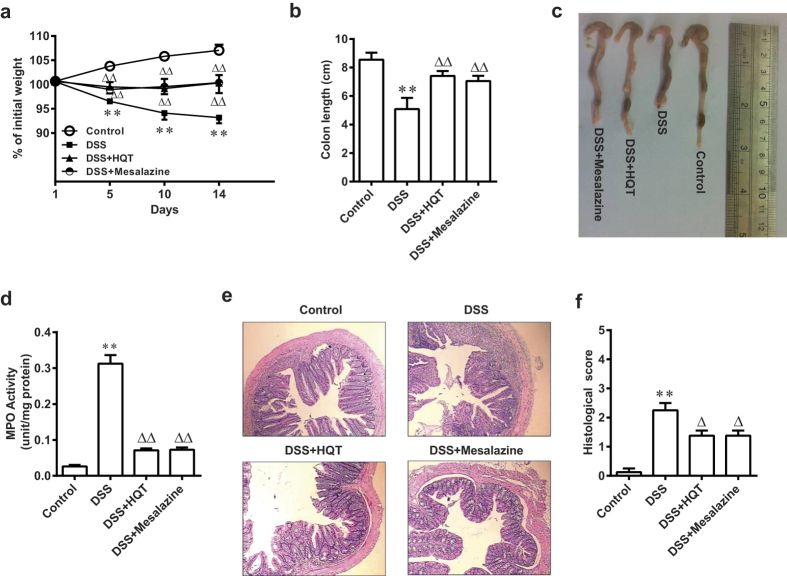
HQT ameliorates DSS-induced acute colitis in mice. Mice were administered regular water (control) or 3.5% DSS for 7 days followed by treatment with HQT for 7 days. (**a**) Body weight, as a percent of starting weight, assessed daily. (**b,c**) Colon length, measured at day 14 after treatment. (**d**) Colonic MPO activity analysis. The results are expressed as units of MPO activity per mg of protein. (**e,f**) Representative H&E-stained distal colonic sections and histological scores, assessed at day 14 after treatment (magnification, x100). Results are expressed as means ± SD of three independent experiments; n = 8 mice per group. ^*^p < 0.05, ^**^p < 0.001 *vs.* the control group; ^△^*p* < 0.05, ^△△^*p* < 0.001 *vs*. DSS-treated mice. MPO, myeloperoxidase.

We used the DSS-induced acute colitis mouse model (mice treated with 3.5% DSS for 7 days) to evaluate the anti-inflammatory effect of HQT in the mouse colon. Mice treated with DSS alone exhibited progressive weight loss compared to control mice, whereas mice that received HQT continued to gain weight (Fig. 1a). Moreover, shortening of the colon and increased myeloperoxidase (MPO) activity, which are indicators of colitis^13^, were clearly observed in mice with DSS-induced colitis. These changes were significantly ameliorated with HQT or mesalazine treatment (Fig. 1b,d). Histology confirmed the clinical signs of colitis by demonstrating the characteristic pattern of inflammation, including reactive epithelial atypia and architectural distortion. The tissue damage was attenuated in HQTor mesalazine treated mice (Fig. 1e,f). These results indicate a protective role of HQT during the progression of acute colitis.

### HQT has a protective effect in a DSS-induced chronic colitis model

**Figure 2:**
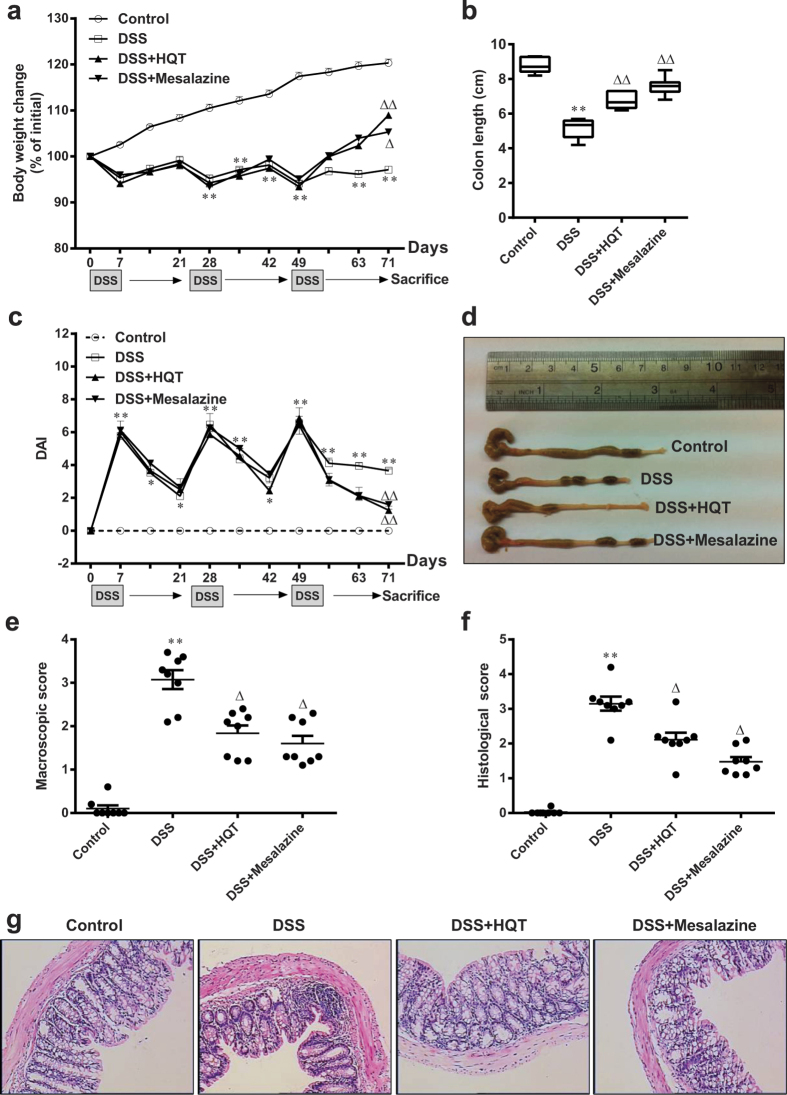
HQT ameliorates DSS-induced chronic colitis. Mice received three cycles of DSS treatment, each cycle consisting of 7 days of 2.5% DSS in drinking water followed by 14 days of tap water, followed by treatment with HQT for 7 days. (**a**) Body weight, as a percent of starting weight, assessed throughout the recurring DSS-induced colitis time course every 7 days. (**b,d**) Colon length, measured at day 71. (**c**) DAI reflecting weight loss, loose stool consistency and amount of blood present in stool and rectum, assessed throughout the recurring DSS-induced colitis time course every 7 days. (**e**) Macroscopic scores, assessed at day 71 after HQT treatment. (**f**) Histological score, assessed at day 71 after HQT treatment. (**g**) H&E staining of representative colon cross-sections on day 71 after HQT treatment (magnification, x100). Results are expressed as means ± SD of three independent experiments; n = 8 mice per group.^*^p < 0.05, ^**^p < 0.001 *vs.* the control group; ^△^*p* < 0.05, ^△△^*p* < 0.001 *vs*. DSS-treated mice. DAI, disease activity index.

We next assessed the effect of HQT in a chronic colitis mouse model created by administering DSS (2.5%) in drinking water for 7 days followed by a 14-day break for a total of three cycles. Body weight and disease activity index (DAI) were recorded every 7 days. DSS-treated mice showed a marked increase in inflammatory response compared to control mice, as indicated by body weight loss and increased DAI (Fig. 2a,c), lower colon length (Fig. 2b,d), more severe inflammation on histological analysis (Fig. 2g) and increased macroscopic and histological scores (Fig. 2e,f). The colonic inflammation was markedly ameliorated in mice that received HQT or mesalazine for 1 week at the end of the third cycle of DSS administration. HQT treatment was significantly associated with less pronounced weight loss and colon shortening, reduced DAI and histological damage and improved histological and macroscopic scores. These observations indicate that HQT has a significant protective effect on the colon of mice with DSS-induced recurring colitis.

### HQT decreases colonic cytokines expression and chemokines production in DSS-induced acute and chronic colitis models

**Figure 3:**
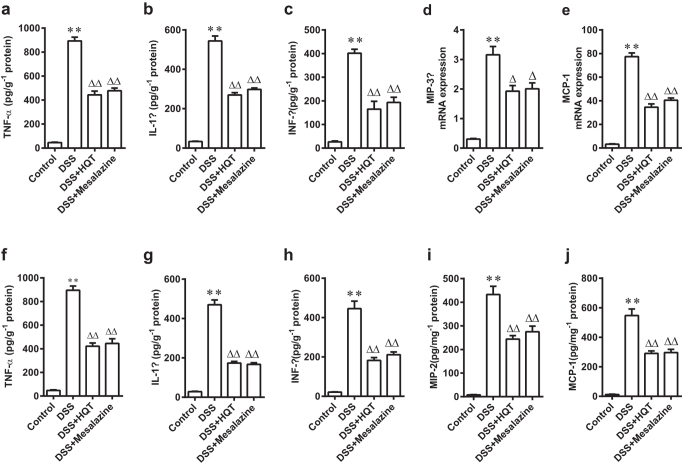
HQT inhibits pro-inflammatory cytokines and chemokines production in colon tissues from mice with DSS-induced acute and chronic colitis. Mice were administered regular water (control) or 3.5% DSS for 7 days followed by treatment with HQT for 7 days. (**a–c**) The protein levels of inflammation-related cytokines TNF-α, IL-1β and IFN-γ in colonic homogenate, determined by ELISA. (**d,e**) Expression of mRNA of inflammatory chemokines MIP-3α and MCP-1 determined by real-time PCR. Results are expressed as means ± SD of three independent experiments; n = 8 mice per. ^*^p < 0.05, ^**^p < 0.001 *vs*. the control group; ^△^*p* < 0.05, ^△△^*p* < 0.001 *vs*. DSS-treated mice. Mice received three cycles of DSS treatment (2.5%), each cycle consisting of 7 days of water containing DSS followed by 14 days of tap water, followed by treatment with HQT for 7 days. (**f–h**) Protein levels of inflammation-related cytokines TNF-α, IL-1β and IFN-γ in colonic homogenates, determined by ELISA. (**i,j**) Protein levels of inflammatory chemokines MIP-2 and MCP-1, determined by ELISA. Results are expressed as means ± SD of three independent experiments; n = 8 mice per group. ^*^p < 0.05, ^**^p < 0.001 *vs.* the control group; ^△^*p* < 0.05, ^△△^*p* < 0.001 *vs*. DSS-treated mice. TNF, tumour necrosis factor; IL, interleukin; IFN, interferon; ELISA, enzyme-linked immunosorbent assay; real-time PCR, Real-time polymer chain reaction.

It is now well accepted that multiple cytokines and chemokines, especially TNF-α, interleukin-1β (IL-1β), interferon-γ (IFN-γ), MIP-3α, MCP-1 and MIP-2^14^,^15^, play an indispensable role in the development and progression of IBD. Enzyme-linked immunosorbent assay (ELISA) revealed lower protein levels of pro-inflammatory cytokines, including TNF-α, IL-1β and IFN-γ, in colon tissues of mice with DSS-induced acute and chronic colitis that received HQT (Fig. 3a–c,f–h). The increased expression of chemokines MIP-3α, MCP-1 and MIP-2 in the colon demonstrated by real-time polymerase chain reaction (PCR) or ELISA analysis in DSS-induced acute and chronic colitis models was reduced significantly by HQT treatment (Fig. 3d,e,i and j). These results suggest that HQT may play a direct role in regulating the inflammatory response to DSS-induced injury.

### HQT inhibits expression of pro-inflammatory cytokines in LPS-stimulated RAW264.7 cells

**Figure 4:**
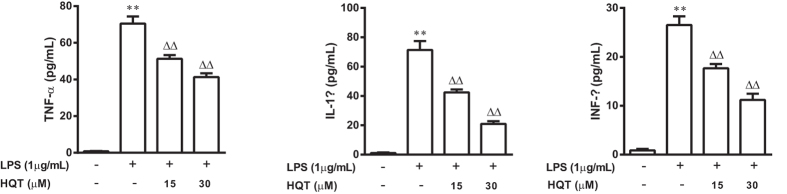
HQT decreases pro-inflammatory cytokines production in LPS-stimulated RAW264.7 cells. RAW264.7 cells were stimulated with LPS (1 μg/mL) with or without HQT at various concentrations (15 and 30 μM) for 24 hours. At the end of incubation, production of cytokines was determined using ELISA. (**a**) TNF-α, (**b**) IL-1β and (**c**) INF-γ. The results are expressed as means ± SD of three independent experiments. *p < 0.05, **p < 0.001 *vs*. the control cells; ^△^*p* < 0.05, ^△△^*p* < 0.001 *vs*. the LPS-stimulated cells. LPS, lipopolysaccharides; ELISA, enzyme-linked immunosorbent assay; TNF, tumour necrosis factor; IL, interleukin.

Pro-inflammatory cytokines and mediators such as TNF-α, IL-1β and INF-γ are key factors involved in regulating inflammatory response^16^. *In vitro* tests demonstrated significant increases in the levels for TNF-α, IL-1β and INF-γ in macrophages stimulated with LPS (1 μg/mL for 24 h) compared to control macrophages. Pre-treatment with HQT (15 and 30 μM) significantly reduced TNF-α, IL-1β and INF-γ secretion from LPS-stimulated macrophages in a dose-dependent manner (Fig. 4a–c).

### HQT suppresses NF-κB activation in LPS-stimulated RAW264.7 cells and mice with DSS-induced acute colitis

**Figure 5:**
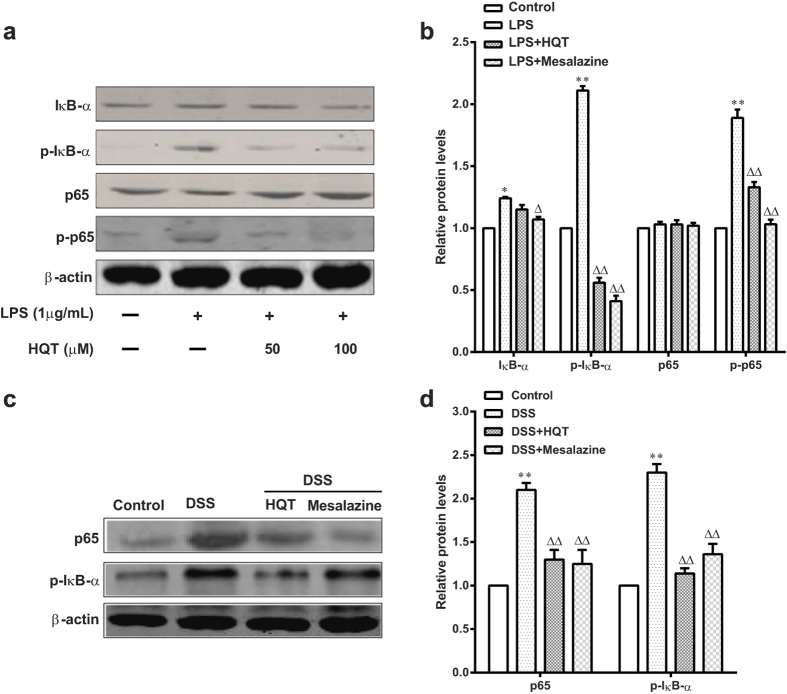
HQT down-regulates NF-κB pathway *in vitro* and *in vivo*. *In vitro* study: RAW264.7 cells were stimulated with LPS (1 μg/mL) with or without HQT at various concentrations (15 and 30 μM) for 24 hours. (**a,b**) Protein levels of IκBα, p-IκBα, p65 and p-p65 measured by Western blotting. The gels were run under the same experimental conditions. Densitometric analysis was performed to determine the relative ratios of each protein. The results are expressed as means ± SD of three independent experiments. *p < 0.05, **p < 0.001 *vs*. the control cells; ^△^*p* < 0.05, ^△△^*p* < 0.001 *vs*. the LPS-stimulated cells. *In vivo* study: Mice were administered regular water (control) or 3.5% DSS for 7 days followed by treatment with HQT for 7 days. (**c,d**) The protein levels of p-IκBα and p65 measured by Western blotting. The gels were run under the same experimental conditions. Densitometric analysis was performed to determine the relative ratios of each protein. Results are expressed as means ± SD of three independent experiments; n = 8 mice per group. ^△^*p* < 0.05, ^△△^*p* < 0.001 *vs*. DSS-treated mice. LPS, lipopolysaccharides.

Nuclear factor-κB (NF-κB) is activated by DSS treatment and plays an important role in intestinal inflammation^17^,^18^,^19^. *In vitro* studies showed that levels of IκB-α, p-IκB-α, p65, and p-p65 increased significantly after LPS stimulation. However, treatment with HQT significantly inhibited LPS-induced stimulation of IκB-α, p-IκB-α, and p-p65 (Fig. 5a,b). Although we observed a pronounced NF-κB activation in colons of mice with DSS-induced colitis, the expression of p65 and p-IκB-α in colonic mucosa was significantly attenuated by HQT treatment (Fig. 5c,d). Therefore, HQT may down-regulate the secretion of pro-inflammatory cytokines by macrophages through suppression of NF-κB activation.

### HQT maintains intestinal epithelial cell homeostasis in mice with DSS-induced acute colitis

**Figure 6:**
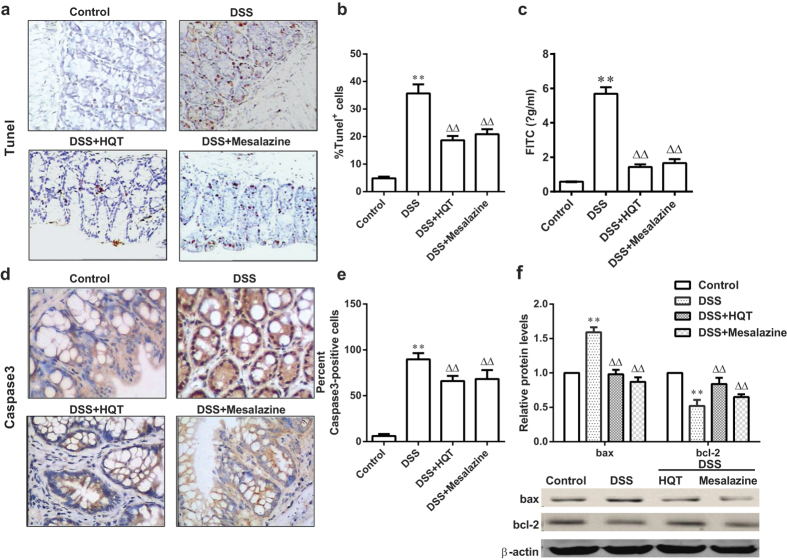
HQT attenuates epithelial cell apoptosis and intestinal permeability in DSS-induced acute colitis. Mice were administered regular water (control) or 3.5% DSS for 7 days followed by treatment with HQT for 7 days. (**a**) TUNEL assay for apoptosis (magnification, x200). (**b**) The percent of TUNEL-positive cells. (**c**) Intestinal permeability assessed by measuring serum FITC-Dextran levels 4 hours after administration. (**d**) Immunohistochemistry analysis of caspase-3 in colon sections (magnification, ×200). (**e**) Graphical representation of Caspase-3 positive cells in the mid-colon. (**f**) Western blotting analyses and densitometric quantitation of colonic mucosal levels of bax and bcl-2 proteins. The gels were run under the same experimental conditions. Results are expressed as mean ± SD of three independent experiments; n = 8 mice per group. ^*^p < 0.05, ^**^p < 0.001 *vs*. the control group; ^△^*p* < 0.05, ^△△^*p* < 0.001 *vs*. DSS-treated mice. TUNEL, terminal deoxynucleotidyl transferase dUTP nick end labelling; FITC, fluorescein isothiocyanate.

To understand how HQT exerts its protective effects on intestinal epithelial cell homeostasis, we analysed cell apoptosis, permeability, tight junction proteins expression and proliferation. TUNEL staining in mice with DSS-induced colitis demonstrated abundant apoptotic colonic epithelial cells. The number of these cells was markedly reduced after HQT treatment for 7 days (Fig. 6a,b). Immunohistochemistry revealed that the expression of caspase-3-positive cells in the colon was reduced after HQT treatment (Fig. 6d,e). Furthermore, results of Western blotting showed that the expression of bax (a pro-apoptotic protein) in the DSS group was elevated compared to that in the control group, whereas HQT treatment clearly reduced bax protein expression levels (Fig. 6f). The levels of bcl-2 (an anti-apoptotic protein) were reduced in DSS-treated mice compared to the controls. HQT treatment stimulated bcl-2 expression in these animals (Fig. 6f).

**Figure 7:**
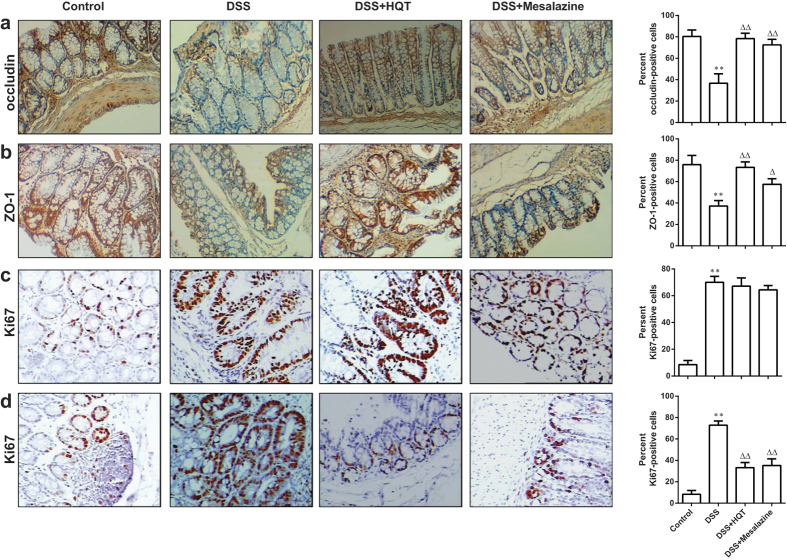
HQT regulates epithelial proliferation in the colonic mucosa of mice with DSS-induced acute and chronic colitis. Mice were administered regular water (control) or 3.5% DSS for 7 days followed by treatment with HQT for 7 days. (**a–c**) Immunohistochemical analysis of tight junction proteins occluding, ZO-1 and proliferating cells detected based on Ki67 in colon sections in DSS-induced acute colitis mice. (magnification, ×200). Graphical representation of the percentage of occluding, ZO-1 and Ki67-positive cells in the mid-colon. Results are expressed as means ± SD of three independent experiments; n = 8 mice per group. ^*^p < 0.05, ^**^p < 0.001 *vs*. the control group; ^△^*p* < 0.05, ^△△^*p* < 0.001 *vs*. DSS-treated mice. Mice received three cycles of DSS treatment (2.5%), each cycle consisting of 7 days of water containing DSS followed by 14 days of tap water, followed by treatment with HQT for 7 days. (**d**) Immunohistochemical analysis of proliferating cells detected based on Ki67 in colon sections in mice with DSS-induced chronic colitis (magnification, ×200). Graphical representation of the percentage of Ki67-positive cells in the mid-colon. Results are expressed as means ± SD of three independent experiments; n = 8 mice per group. ^*^p < 0.05, ^**^p < 0.001 *vs*. the control group; ^△^*p* < 0.05, ^△△^*p* < 0.001 *vs*. DSS-treated mice.

Intestinal permeability, an indicator of epithelial barrier integrity, was measured quantitatively by feeding mice with fluorescein isothiocyanate (FITC)-dextran. Serum fluorescence was quantified 4 hours after the gavage. The serum levels of FITC-conjugated dextran were markedly increased in mice treated with DSS (Fig. 6c). These levels were markedly reduced by HQT treatment. To further confirm that HQT regulates intestinal barrier function, we analyzed the expression of tight junction proteins occluding and zonula occludens-1 (ZO-1) in the colon of mice with DSS-induced colitis. As expected, the expression of occludin and ZO-1 was significantly increased in the colon by HQT (Fig. 7a,b). These data indicate that HQT restores the integrity of intestinal epithelial barrier in DSS-treated mice.

Ki67 is a well-established marker of proliferating epithelial cells that plays a central role in inflammation. Therefore, Ki67–immunoreactive cell numbers were determined in colonic tissue as a marker of epithelial cell proliferation. Epithelial cells of tissue sections from control mice exhibited normal levels of specific staining for Ki67, whereas strong immunostaining for Ki67 was present in both models of DSS-induced colitis. Interestingly, treatment with HQT following DSS administration significantly reduced the intensity of Ki67 immunostaining in the model of chronic colitis (Fig. 7d) but not in the model of acute colitis (Fig. 7c).

### HQT administration induces CD4+CD25+Foxp3+ regulatory T cells in mice with DSS-induced chronic colitis

**Figure 8:**
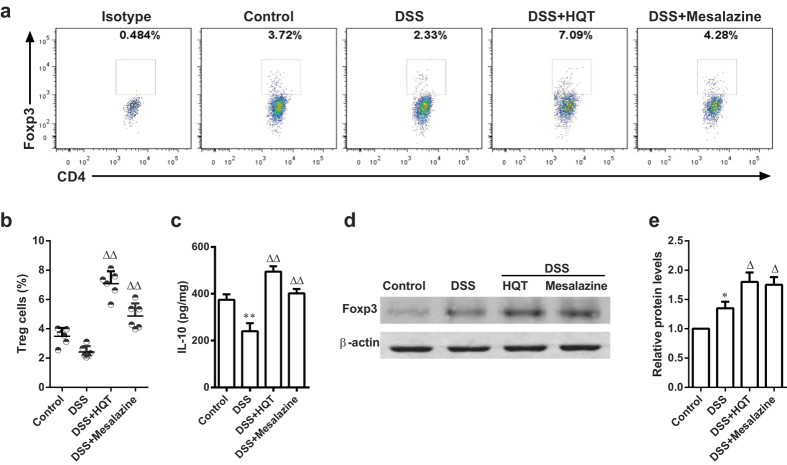
HQT induces Treg cells in mice with DSS-induced chronic colitis. Mice received three cycles of DSS treatment (2.5%), each cycle consisting of 7 days of water containing DSS followed by 14 days of tap water, followed by treatment with HQT for 7 days. LPMCs were isolated from each group and subjected to intracellular Foxp3 staining. (**a**) The frequency of Treg cells (CD4+CD25+Foxp3+) determined by flow cytometry. Numbers indicate percentages of Foxp3-expressing CD4+CD25+T cells in each quadrant. (**b**) Quantitative analysis of the frequency and total number of Treg in LPMCs. (**c**) IL-10 in colonic homogenate determined by ELISA. (**d,e**) Western blot analysis of Foxp3 protein expression in the colon. The gels were run under the same experimental conditions. Densitometric analysis to determine the relative ratios of Foxp3 protein. Results are expressed as mean ± SD of three independent experiments; n = 8 mice per group. ^*^p < 0.05, ^**^p < 0.001 *vs*. the control group; ^△^*p* < 0.05, ^△△^*p* < 0.001 *vs*. DSS-treated mice. Treg, regulatory T cells; LPMC, lamina propria mononuclear cells; ELISA, enzyme-linked immunosorbent assay; IL, interleukins.

Regulatory T (Treg) cells expressing transcription factor Foxp3 play a crucial role in the immune system by preventing dysregulated inflammatory responses following chronic immune stimulation^20^. Studies of mouse models and human patients suggest that absence of Treg cells exacerbates intestinal inflammation^21^,^22^. We sought to determine whether protection by HQT was associated with Treg cell changes in the DSS-induced chronic colitis model. Flow cytometry experiments revealed that the frequency of Treg cells in lamina propria mononuclear cells (LPMCs) was slightly reduced in DSS-treated mice compared to that in control mice. The Treg cells frequency was significantly increased by treating mice with DSS-induced colitis with HQT (Fig. 8a,b). Furthermore, protein levels of anti-inflammatory cytokine IL-10 in the colon were lower in the DSS group than in the control group as determined by ELISA. There was a significant increase in IL-10 levels following treatment with HQT (Fig. 8c). Moreover, Western blotting revealed markedly elevated levels of Foxp3 protein in the colon after HQT treatment. Taken together, these data suggest that HQT might contribute to the generation of Treg cells in DSS-induced chronic colitis.

### Qualitative and quantitative analysis of HQT components

Active components of HQT were identified by high performance liquid chromatography (HPLC), with paeoniflorin, baicalin and baicalein used as reference standards. By comparing major peaks on HQT chromatograms with the standard peaks, we found that paeoniflorin, baicalin and baicalein are three distinct components of HQT. Paeoniflorin is derived from *Paeonia lactiflora* Pall, whereas baicalin and baicalein originate mainly from *Scutellaria baicalensis* Georgi (Fig. S1). Paeoniflorin, baicalin and baicalein represented 11.38%, 4.23% and 6.73%, respectively, of the total material (Table. S1).

## Discussion

In this study, we demonstrated for the first time that the effectiveness of HQT in treating DSS-induced acute and chronic colitis exceeds that of mesalazine. Treatment with HQT reduced body weight loss and colon shortening, ameliorated mucosal inflammation and led to clinical improvement in mice with DSS-induced acute and chronic colitis, suggesting therapeutic potential. Mechanistically, we show that DSS-induced nuclear NF-κB signalling is inhibited by HQT. Furthermore, our findings revealed that HQT plays a critical role in the maintenance of intestinal integrity and homeostasis by significantly affecting cell proliferation and apoptosis and intestinal permeability. Our data also suggest a crucial role of HQT in the induction of Treg cells. Therefore, HQT exerts multiple effects that regulate intestinal homeostasis and intestinal epithelial barrier function and play a critical role in controlling the inflammatory and immune response occurring during experimental colitis.

The therapeutic effect of HQT was demonstrated *in vivo* using mouse models of DSS-induced acute and chronic colitis. These widely employed colitis models are characterised by weight loss, bloody diarrhoea, epithelial damage, increased production of inflammatory mediators and immune cell infiltration of colonic mucosa^23^,^24^. However, the effect of HQT on DSS-induced colitis has not been studied so far. In this study, DSS-exposed mice displayed profound and sustained weight loss, colonic shortening and higher histological scores for colonic inflammation than control mice. HQT treatment significantly ameliorated these clinical signs. Furthermore, histological analysis of colon tissues showed less damage to the crypt architecture in HQT-treated mice than in mice treated with DSS only. Treatment with HQT also resulted in microscopic amelioration of intestinal inflammation, as demonstrated by the reduction of MPO activity, a marker of mucosal neutrophil activation. These results are in agreement with those of previous studies, including our study that demonstrated alleviation of inflammation in TNBS-induced murine colitis by HQT^11^,^12^. It has been reported that agents such as mesalazine, olsalazine and sulfasalazine are effective in preventing colitis in this animal model^25^. The results of the current and previous studies suggest that HQT has a protective effect in DSS-induced colitis equal to or even greater than that of mesalazine (used as a reference in the present work) or sulfasalazine^11^,^12^. Hence, HQT may be a promising candidate for the treatment of colitis.

Multiple cytokines have been implicated in the initiation, progression and development of murine and human IBD^16^. It is known that chemokines such as MIP-3α, MCP-1 and MIP-2 are expressed specifically in the inflamed areas of the colon in patients with IBD^14^,^15^. It is also known that HQT treatment decreases pro-inflammatory cytokine levels, including TNF-α, IL-1β and IL-6, in TNBS-induced colitis in rats^11^. Our current results obtained in mice with DSS-induced acute and chronic colitis further confirm this inhibitory effect of HQT on cytokines and chemokines. In our animal model, the levels of cytokines such as TNF-α, IL-1β and IFN-γ in the colon decreased following the administration of HQT. In addition, the expression of MIP-3α, MCP-1 and MIP-2 was significantly down-regulated in the colon of mice with DSS-induced acute and chronic colitis after HQT treatment. The anti-inflammatory activity of HQT was confirmed *in vitro* in LPS-stimulated RAW 264.7 cells. Thus, the production of TNF-α, IL-1β and IFN-γ in culture supernatants of LPS-stimulated cells was markedly reduced following HQT treatment. These results suggest that HQT may suppress the secretion of pro-inflammatory cytokines by macrophages, which may explain its anti-inflammatory effect.

We further analysed the molecular mechanism underlying the HQT-mediated suppression of colonic inflammation. NF-κB is a nuclear transcription factor that regulates the expression of genes encoding pro-inflammatory mediators, which play a key role in initiating bowel inflammation in CD and UC, as well as in models of experimental colitis^19^. It has been reported that mice with DSS- and TNBS-induced colitis and IL-10 knockout mice are characterised by NF-κB activation, which in turn induces transcription of pro-inflammatory cytokines and chemokines^26^,^27^. Our study is the first to demonstrate that HQT inhibits the expression of p65 and p-IκB-α in the colonic mucosa of mice with DSS-induced colitis. Furthermore, HQT treatment was associated with a concomitant reduction in the levels of LPS-induced IκB-α, p-IκB-α and p-p65. These results indicate that HQT may reduce the severity of colitis by effectively down-regulating the secretion of pro-inflammatory cytokines by macrophages via inactivation of the NF-κB signalling pathway.

Aberrant intestinal epithelial cell homeostasis plays a central role in the pathophysiology of IBD. A balance of epithelial cell apoptosis and proliferation determines normal tissue homeostasis. Increased apoptosis in gut epithelial cells compromises the mucosal barrier, leading to colonic inflammation. This has been reported in patients with UC and CD^28^,^29^,^30^, as well as in murine models of colitis^31^,^32^. Increased intestinal permeability is common in first-degree relatives of patients with IBD, suggesting that barrier dysfunction may be an early defect^33^. Our results of TUNEL staining, along with caspase-3, bax and bcl-2 levels, demonstrated that colonic epithelial cell apoptosis was significantly down-regulated by HQT. Multiple tight junction proteins are involved in maintaining the physical barrier by strongly limiting the intestinal permeability^34^. Our data are consistent with an effect of HQT on mucosal tight junctions. It was found that the administration of HQT increased occludin and ZO-1 expression in DSS-induced colitis model. The DSS-induced colitis model is characterised by epithelial injury during exposure to DSS followed by epithelial repair and increased epithelial proliferation upon DSS withdrawal^35^,^36^. Increased proliferation of IECs is thought to be an important defensive mechanism that allows expelling of invasive intestinal pathogens. However, the tumour-promoting effect of chronic inflammation is widely recognised to be related to the stimulation of excessive cell proliferation^37^. Treatment with HQT did not induce any significant differences in the expression of the proliferative marker Ki67 in mice with DSS-induced acute colitis. Interestingly, treatment with HQT following DSS administration significantly reduced the proliferation rate in mice with DSS-induced chronic colitis. We speculate that suppression of excessive cell proliferation by HQT in chronic colitis may be important for prevention of colitis-associated cancer. These findings suggest that HQT treatment may improve intestinal epithelial cell homeostasis in DSS-induced injury.

Our data also suggest a role for HQT in immune regulation and protection from experimental colitis. Treg cells are specialised CD4+T cells that play a crucial role in the maintenance of immune homeostasis and suppression of inflammatory response^38^. Treg cells suppress effector T cell responses and have a major protective role in experimental colitis. This role is realised via production and release of anti-inflammatory cytokines such as IL-10 and transforming growth factor-β (TGF-β)^39^. In our study, HQT treatment was associated with a significant increase in the number of CD4+CD25^+^ Foxp3^+^ Treg cells and in the expression level of Foxp3 in the colon of mice with DSS-induced chronic colitis. In agreement, the levels of IL-10 were higher in the colon homogenate supernatants of mice in the HQT group than in the DSS group. These data indicate that HQT may control colitis by inducing Treg cells.

Impaired intestinal epithelial homeostasis has been shown to increase susceptibility to immune-mediated inflammatory disorders, including IBD. These disorders are characterised by elevated pro-inflammatory cytokines, exaggerated immune response and increased apoptosis, disrupting the homeostasis of the protective intestinal epithelial monolayer^2^,^40^. Although we found that HQT treatment suppresses the development of colitis and regulates immune response, it remains unclear whether regulation of immune response is the key mechanism by which HQT alleviates colitis. HQT may also directly regulate non-immunological factors that are critical for the pathogenesis of DSS-induced colitis, such as the impaired intestinal epithelial homeostasis and intestinal epithelial barrier function, thereby protecting against IBD. Furthermore, because TCM formulae are based on a multicomponent, multitarget approach with the ultimate goal of restoring the balance in the human body, the effect of HQT on colitis may be mediated by multiple molecules and pathways. More studies are required to define the precise cellular and molecular mechanisms underlying the anti-inflammatory effect of HQT in DSS-induced colitis.

## Conclusions

This study provides valuable new insights into the protective effect of a TCM formula, HQT, in DSS-induced colitis. Our results strongly suggest that HQT exerts a protective effect on the colon in DSS-induced colitis by targeting multiple cellular pathways. The key mechanism of HQT’s mode of action is regulation of colonic epithelial homeostasis and reduction of colonic epithelial permeability, which in turn strengthens intestinal mucosal barrier function and regulates mucosal immune activation and inflammation. Interestingly, our data indicate that HQT is more efficient in controlling colitis than mesalazine. In addition, HQT effectively ameliorated not only DSS-induced acute colitis but also chronic colitis. Therefore, HQT is a potential candidate for future clinical applications in IBD.

## Methods

### Mice

Pathogen-free male and female C57BL/6 mice were purchased from the Laboratory Animal Center of SunYat-Sen University (Guangzhou, China). All mice were housed in cages under laboratory conditions (18–25 °C, 60–70% humidity, natural light) with free access to food and water. All experiments were approved by the Animal Experimentation Ethics Committee at Guangdong Medical University, and the animal study protocol was carried out in accordance with the approved guidelines.

### Composition and preparation of HQT

Herbal materials of *Scutellaria baicalensis* Georgi. (90 g), *Paeonia lactiflora* Pall. (60 g), *Glycyrrhiza uralensis* Fisch. (60 g) and *Ziziphus jujuba* Mill. (60 g) were purchased from Tongren Tang drug store (Dongguan, Guangdong, China). All herbal materials were accredited by a pharmacologist, Professor Jiantao Lin. HQT was extracted according to the standard methods recommended by the Chinese Pharmacopoeia (2010). Briefly, HQT formulae were soaked in distilled water for 30 min and then boiled in 10 volumes of water (v/w) for 1 hour. After filtration, the residue was extracted in 5 volumes of boiling water for 1 hour, and filtered. Thereafter, the two extracts were combined in airtight containers and stored at 4 °C. A single HQT batch was produced. The solution was diluted with distilled water to appropriate concentrations for animal treatment.

HQT for cell culture assays was prepared by extracting the herbal juice twice in ten-fold volume of 70% ethanol for 1 hour each time. The combined extracts were concentrated under vacuum, filtered through a 0.22 μm membrane filter, and dissolved in dimethyl sulfoxide (DMSO) (1 g/mL) for use in *in vitro* experiments.

HPLC profiling was performed using an Agilent 1200 liquid chromatography system (Agilent Technologies Co., Ltd., USA) equipped with a quaternary pump, auto-sampler, column temperature controller, online degasser and a G1314B VWD detector. Separation was performed at a flow rate of 1.0 ml/min and temperature of 40 °C on an Eclipse plus C18 column (150 × 4.6 mm, 5 μm). The mobile phase consisted of acetonitrile (A) and 0.2% aqueous acetic acid (B). The following linear gradient elution protocol was used: 0–10 min: 5% A, 10–15 min: 5–20% A, 15–30 min: 20–25% A, 30–40 min: 25–30% A, 40–55 min: 30–75% A, 55–60 min: 75–5% A. The detector was set at 230 nm.

### Models of experimental colitis and treatment

Acute colitis was induced in C57BL/6 mice by administration of 3.5% (w/v) DSS (36–50 kDa, MP Biomedicals) in drinking water for 7 consecutive days *ad libitum* as described previously^41^. For inducing chronic colitis, mice were exposed to 3 cycles consisting of 7 days of 2.5% DSS administration in drinking water followed by 14 days of DSS-free distilled water (DSS-free interval)^42^. Control groups received normal drinking water without DSS. Mice were administered HQT in distilled water at 4.5 g/kg of body weight. Mesalazine from Ethypharm (Houdan, France) was used at a dose of 100 mg/kg as a vehicle control. HQT and mesalazine were administered by oral gavage twice daily for one week starting from 24 h after colitis induction. Control mice had free access to tap water.

### Cell culture and treatment

The RAW 264.7 mouse macrophage cell line was purchased from the Cell Bank of Shanghai Institutes of Biological Sciences, Chinese Academy of Sciences (Shanghai, China) and cultured in Dulbecco’s modified Eagle’s medium (DMEM) (HyClone) supplemented with 10% foetal bovine serum (FBS, HyClone), 100 U/ml penicillin and 100 μg/ml streptomycin (HyClone) at 37 °C and 5% carbon dioxide (CO_2_). RAW264.7 cells (1.0 × 10^6^) in 500 μL of medium were added to 48-well plates. After 12 hours, cells were treated with LPS (Sigma-Aldrich) at a concentration of 1 μg/mL, with or without HQT (15 and 30 μM) for 24 hours. Cell culture media were collected, and levels of TNF-α, IL-1β, and INF-γ in supernatants were determined by ELISA according to the manufacturer’s instructions. The expression of p65 and p-IκB-α was detected by Western blot analysis.

### Measurement of colitis severity

The severity of colitis during the acute course was estimated daily by measuring weight loss, rectal bleeding, and stool consistency^43^. The appearance of blood in the stool was measured by stool guaiac test (Beckman Coulter). The DAI was assessed by an investigator blinded to the project using the following evaluation system^44^: body weight loss (0 = no weight loss, 1 = weight loss of 1–5% from baseline, 2 = 5–10%, 3 = 10–20%, 4 = > 20%); stool consistency (0 = well-formed pellets, 2 = pasty and semi-formed stool not adhered to the anus, 4 = diarrhoea adhered to the anus.) and faecal blood score (0 = negative stool haemoccult test; 2 = positive haemoccult; 4 = gross bleeding). For each parameter, a score of 0 to 4 was attributed, leading to a maximal score of 12. The entire colon, from the caecum to the anus, was removed post-mortem, and the colon length was measured as a marker of inflammation.

### Macroscopic scoring

The distal colon was removed, opened longitudinally and gently rinsed with ice-cold phosphate buffer (pH 7.4). Macroscopic damage was assessed by an investigator blinded to the project using a 0–4 colon macroscopic scoring system^45^ with certain modifications: 0 = normal; 1 = hyperaemia without ulcers; 2 = linear ulcer with no significant inflammation; 3 = wide ulcers and necrosis and/or adhesions; 4 = megacolon and/or stenosis and/or perforation.

### Histology and scoring

Colonic segments were fixed in 10% formalin and embedded in paraffin. Sections (5-μm-thick) were stained with haematoxylin & eosin (H&E) in a standard fashion. Histologic scoring was performed by an investigator blinded to the project based on the previously described criteria^44^: 0 = normal; 1 = moderate mucosal inflammation without erosion and ulcer; 2 = severe mucosal inflammation with erosion; 3 = severe mucosal inflammation with ulcer (<1 mm); 4 = severe mucosal inflammation with ulcer (1–3 mm); 5 = severe mucosal inflammation with ulcer (>3 mm).

### Immunohistochemistry

Colonic tissues were fixed in 10% buffered neutral formalin, dehydrated, and paraffin embedded. Sections (5-μm-thick) were deparaffinised and rehydrated, processed by microwave antigen retrieval, and incubated overnight with primary antibodies against Ki67 (Dako), occludin and ZO-1 (Bioss) at 4 °C. They were further incubated with biotinylated secondary antibodies (Vector Labs) and streptavidin–horseradish peroxidase with diaminobenzidine (Vector Labs).

### MPO activity

Colonic tissues of all mice were removed upon dissection, weighed, and immediately flash-frozen. Processing of tissues for MPO activity assessment was performed as described by the manufacturer (Nanjing Jiancheng Bioengineering Institute). The change in absorbance at 490 nm was determined using a spectrophotometer (Benchmark microplate reader, Bio-Rad Labs). Results were expressed in unit/mg tissue.

### Assay of cytokine production in colonic mucosa

Colonic mucosa was cut into pieces and homogenised in ice-cold 100 mM Tris-HCl buffer, pH 7.0, containing a cocktail of protease inhibitors (Beyotime) and supplemented with 1 mM phenylmethanesulfonyl fluoride. The levels of TNF-α, IL-1β and IFN-γ were measured using ELISA kits (R&D) according to the manufacturer’s recommendations.

### Real-time polymerase chain reaction (PCR)

The isolation of total RNA, preparation of cDNA, and amplification of target genes by PCR were carried out following our published protocol^12^. Briefly, total RNA was extracted from the colon using Trizol reagent (Invitrogen) and reversely transcribed. The cDNA was used as a template for real-time PCR with a SYBR green qPCR mixture following the manufacturer’s protocol (Invitrogen). The gene-specific primers were as follows: MCP-1: 5′-CCCAATGAGTAGGCTGGA GA-3′ (forward), 5′-TCCTTCTTGGGGTCAGCACAGAC-3′ (reverse); MIP-3α: 5′-GCTGATCCAAAGCAGAACT-3′ (forward), 5′-CTGGGGTTGAAGGCTTCACC-3′ (reverse). Mean relative gene expression was calculated using the 2^−ΔΔCt^ formula.

### TUNEL staining

TUNEL staining with an ApopTag Kit (Millipore) was performed to detect apoptotic cells in colon sections according to the manufacturer’s instructions. Apoptosis was semi-quantified by assessing TUNEL-positive crypts in 100 randomly selected crypts for each mouse, and the apoptotic index was defined as the percentage of TUNEL-positive cell-containing crypts, as previously described^46^.

### *In vivo* permeability assay

To assess intestinal permeability, food and water were withdrawn for 4 hours, and mice were gavaged with a permeability tracer, FITC-conjugated dextran (Sigma), at a concentration of 0.6 mg/g body weight. Retro-orbital blood samples were collected after 4 hours, and serum fluorescence intensity was measured using a fluorescence spectrophotometer (excitation, 492 nm; emission, 525 nm; Cytofluor 2300 nm). FITC-dextran concentrations were determined from standard curves generated by serial dilution of FITC-dextran.

### Protein extraction and immunoblotting analysis

Total protein was extracted from the mucosal samples of mice and RAW 264.7 cell lysates in radioimmunoprecipitation assay (RIPA) buffer supplemented with 1% protease inhibitor cocktail (Roche Diagnostics) and 1% phosphatase inhibitor (Millipore). Protein concentration in the lysate was determined by the Pierce^TM^ BCA protein assay kit (Thermo). After boiling samples for 10 minutes, equal amounts of protein (50 μg) were separated by sodium dodecyl sulphate-polyacrylamide gel electrophoresis (SDS-PAGE) and electrophoretically transferred to polyvinylidenedifluoride (PVDF) membranes (Millipore). Blocking was done by incubating the membranes for 1 hour at room temperature with 5% non-fat, dry milk diluted in TBS containing 0.1% Tween-20. The membranes were then incubated overnight with primary antibodies against IκB-α, p-IκB-α, p65, p-p65, bax and bcl-2 (Cell Signaling Technology) at 4 °C with gentle shaking. Membranes were then incubated with the secondary antibody for 1 to 2 hours. Protein expression level was analysed by a LI-COR Odyssey Infrared Imaging System. β-actin (Santa Cruz) was used as an internal control.

### Preparation of lamina propria mononuclear cells (LPMCs)

LPMCs were isolated using a modified published method^47^. Briefly, the intestinal mucosa was washed in complete Hank’s balanced salt solution (HBSS) without Ca^2+^ and Mg^2+^, cut into 5-mm pieces and incubated in a medium containing 5 mM ethylenediaminetetraacetic acid (EDTA) (Sigma) and 1 mM dithiothreitol (DTT) (Sigma) at 37 °C for 30 minutes until all crypts and individual epithelial cells were removed. The tissues were digested further in Roswell Park Memorial Institute (RPMI) medium 1640 (Gibco) containing 10% foetal calf serum (FCS) (HyClone), 0.15 g of collagenase type IV (Sigma), and 0.025 g of DNase I (Sigma) in a shaking incubator at 37 °C. The tissue slurry was then passed through a 70-μm cell strainer to remove undigested pieces, centrifuged and resuspended in 40–60% Percoll solution (Amersham Biosciences) density gradient. Cells were frozen in liquid nitrogen for storage at a concentration of 1 × 10^6^ cells/mL until analysis.

### Flow cytometry

Isolated LPMCs were resuspended in phosphate buffered saline (PBS) containing 5% FBS. Foxp3 staining was performed in accordance with the manufacturer’s instructions (eBioscience). Surface staining was performed with FITC-conjugated anti-CD4 and phycoerythrin (PE)-conjugated anti-CD25 antibodies. Cells were then fixed and permeabilised with Fix/Perm buffer, and intracellular staining was performed with APC-conjugated anti-Foxp3 antibodies. The stained cells were analysed by a fluorescence activated cell sorting (FACS) Canto cytometer (BD Bioscience). Data were analysed with FlowJo software, version 7.5 (Tree Star). Isotype-matched antibodies were used as controls.

### Statistics

Data were analysed using unpaired Student’s t-test. For multiple groups, nonparametric Kruskal-Wallis or parametric one-way ANOVA was used. Values were expressed as means ± standard deviation (SD). *P* values < 0.05 were considered to represent statistically significant differences.

## Additional Information

**How to cite this article:** Zou, Y. *et al*. Huangqin-tang ameliorates dextran sodium sulphate-induced colitis by regulating intestinal epithelial cell homeostasis, inflammation and immune response. *Sci. Rep.* **6**, 39299; doi: 10.1038/srep39299 (2016).

**Publisher's note:** Springer Nature remains neutral with regard to jurisdictional claims in published maps and institutional affiliations.

## Supplementary Material

Supplementary Material
