# Supplementary material for: Huangqin-tang ameliorates dextran sodium sulphate-induced colitis by regulating intestinal epithelial cell homeostasis, inflammation and immune response
Source: Sci Rep. 2016 Dec 16;6:39299. doi: 10.1038/srep39299 (PMC5159883; doi:10.1038/srep39299)
Supplement: Supplementary Material [file srep39299-s1.pdf]

Huangqin-tang ameliorates dextran sodium sulphate-induced colitis by regulating intestinal epithelial cell homeostasis, inflammation and immune response

Ying Zou[1,3†], Jiantao Lin[4†], Wenyang Li[1], Zhuguo Wu[5], Zhiwei He[3], Guoliang Huang[3], Jian Wang[3], Caiguo Ye[3], Xiaoyan Cheng[1], Congcong Ding[3], Xuebao Zheng[2*] & Honggang Chi[1*]

[1]Department of Traditional Chinese Medicine, Scientific Research Platform, The Second Clinical Medical College, Guangdong Medical University, Dongguan 523808, China

[2]Mathematical Engineering Academy of Chinese Medicine, Guangzhou University of Chinese Medicine, Guangzhou, 510006, China.

[3]Sino-American Cancer Research Institute, Key Laboratory for Medical Molecular Diagnostics of Guangdong Province, Guangdong Medical University, Dongguan 523808, China

[4]Traditional Chinese Medicine and New Drug Research Institute, Guangdong Medical University, Dongguan 523808, China

[5]The Second Clinical Medical College, Guangdong Medical University, Dongguan 523808, China

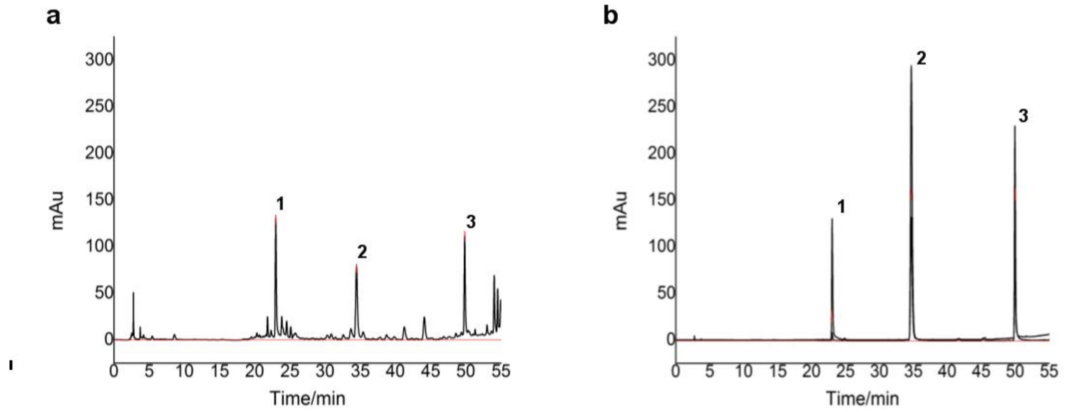

Supplementary Figure 1. HPLC chromatogram of main active components in an aqueous extraction of HQT. (a) aqueous extract of HQT. (b) Standards: 1, paeoniflorin; 2, baicalin; 3, baicalein.

Supplementary Table 1 Characterisation of HQT components by HPLC

Peak no	Component	Molecular formula	Content（%）
1	paeoniflorin	$C_{23}H_{28}0_{11}$	11.38
2	baicalin	$C_{21}H_{18}O_{11}$	4.23
3	baicalein	$C_{15}H_{10}O_5$	6.73
